# Structural Basis for the Recognition of Histone H4 by the Histone-Chaperone RbAp46

**DOI:** 10.1016/j.str.2008.05.006

**Published:** 2008-07-09

**Authors:** Natalia V. Murzina, Xue-Yuan Pei, Wei Zhang, Mike Sparkes, Jose Vicente-Garcia, J. Venkatesh Pratap, Stephen H. McLaughlin, Tom Rolef Ben-Shahar, Alain Verreault, Ben F. Luisi, Ernest D. Laue

**Affiliations:** 1 Department of Biochemistry, 80 Tennis Court Road, Cambridge CB2 1GA, UK; 2 Institut de Recherche en Immunologie et Cancer (IRIC), Université de Montréal, Montréal, Quebec H3T 1J4, Canada

**Keywords:** PROTEINS, DNA

## Abstract

RbAp46 and RbAp48 (pRB-associated proteins p46 and p48, also known as RBBP7 and RBBP4, respectively) are highly homologous histone chaperones that play key roles in establishing and maintaining chromatin structure. We report here the crystal structure of human RbAp46 bound to histone H4. RbAp46 folds into a seven-bladed β propeller structure and binds histone H4 in a groove formed between an N-terminal α helix and an extended loop inserted into blade six. Surprisingly, histone H4 adopts a different conformation when interacting with RbAp46 than it does in either the nucleosome or in the complex with ASF1, another histone chaperone. Our structural and biochemical results suggest that when a histone H3/H4 dimer (or tetramer) binds to RbAp46 or RbAp48, helix 1 of histone H4 unfolds to interact with the histone chaperone. We discuss the implications of our findings for the assembly and function of RbAp46 and RbAp48 complexes.

## Introduction

The nucleosome is the fundamental structural unit of chromatin in all eukaryotic cells. The core of the nucleosome is an octameric histone complex, comprising two H3/H4 heterodimers that associate to form a tetramer and that then bind two H2A/H2B dimers. Nucleosomes convey chemically encoded information either through covalent modifications or the incorporation of variant histone isoforms. They are made during replication, when histone octamers must be assembled and incorporated into DNA de novo. Histone chaperones are essential components of the machinery that aides the assembly of nucleosomes during replication and recruits the enzymes needed to mark the histones covalently. In addition, histone chaperones are also found in many chromatin-associated complexes, where they often work in conjunction with enzymes that remodel chromatin structure by repositioning nucleosome cores in an ATP-dependent manner.

RbAp46 and RbAp48 are highly homologous histone chaperones that play key roles in protein complexes that function to establish and maintain chromatin structure. They are integral subunits of protein complexes that either add or remove acetyl moieties from the ɛ-amino groups of lysine residues on the flexible N-terminal tail of histone H4, and they are also key components of histone methylases. RbAp46 binds to and enhances the activity of the type B histone acetyltransferase HAT1, an enzyme that acetylates histone H4 specifically at lysine residues 5 and 12 prior to their incorporation into nucleosomes during replication (Parthun et al., 1996; Verreault et al., 1998). This specific pattern of acetylation of newly synthesized histone H4 is conserved from yeast to humans (Benson et al., 2006). The importance of this modification is indicated by the crucial role HAT1 plays in the cellular resistance to genotoxic agents (Barman et al., 2006; Benson et al., 2007). RbAp48, on the other hand, is an evolutionarily conserved subunit of the chromatin assembly factor-1 (CAF-1) complex, where it associates with two other subunits, known as p150 and p60 in human cells. Like RbAp46, RbAp48 also contributes to a large number of other protein complexes within the cell, and they are sometimes both found in the same protein complex. Intriguingly, although RbAp46 and RbAp48 are 90% identical at the amino acid sequence level, in both human and yeast cells RbAp46 is found exclusively in complexes with HAT1, whereas RbAp48 is instead found in the CAF-1 complex (Parthun et al., 1996; Kaufman et al., 1997; Verreault et al., 1996, 1998). Both RbAp46 and RbAp48 are found in the catalytic cores of histone deacetylase (HDAC) complexes that promote transcriptional repression of target genes, including those repressed by the retinoblastoma tumor suppressor protein (Korenjak et al., 2004; Nicolas et al., 2000; Zhang et al., 1999). The other components of the core HDAC complex are HDAC1 and HDAC2 (Zhang et al., 1999). This core HDAC complex associates with other proteins in both the Sin3A HDAC complex (Vermaak et al., 1999; Zhang et al., 1998) and the nucleosome remodeling and HDAC complex, NuRD (Wade et al., 1998; Zhang et al., 1999). In addition, both RbAp46 and RbAp48 are found as components of two distinct EZH2/EED complexes that methylate either histone H3 lysine 27 or histone H1 lysine 26 to promote epigenetic silencing of regulatory genes during development (Kuzmichev et al., 2004). Moreover, both proteins are also components of the ATP-dependent nucleosome remodeling factor (NURF), which has been implicated in transcriptional regulation (Barak et al., 2003; Martinez-Balbas et al., 1998). In *Drosophila*, p55 is the sole ortholog of RbAp46/48 and, in addition to being a subunit of CAF-1, p55 also forms a complex with histone H4 and an essential centromeric variant of histone H3, known as CID in *Drosophila* or CENP-A in mammals (Furuyama et al., 2006). Finally, compelling evidence indicates that orthologs of p55 in *S*. *pombe* (Mis16) and human cells (RbAp48) are required for deposition and/or retention of CENP-A into centromeric chromatin (Hayashi et al., 2004). This is puzzling because CAF-1 deposits histone H3/H4 throughout the genome, whereas the p55/CENP-A/H4 complex is likely specific for centromeres.

We report here the crystal structure of human RbAp46 bound to histone H4. Our results suggest that when the histone H3/H4 dimer (or tetramer) binds RbAp46, helix 1 of histone H4 unfolds and adopts a different conformation than is observed in either the nucleosome or in the complex with ASF1, another histone chaperone. We discuss the implications of our findings for the orchestrated assembly and function of RbAp46 and RbAp48 complexes.

## Results

### Overall Structure

**Figure 1:**
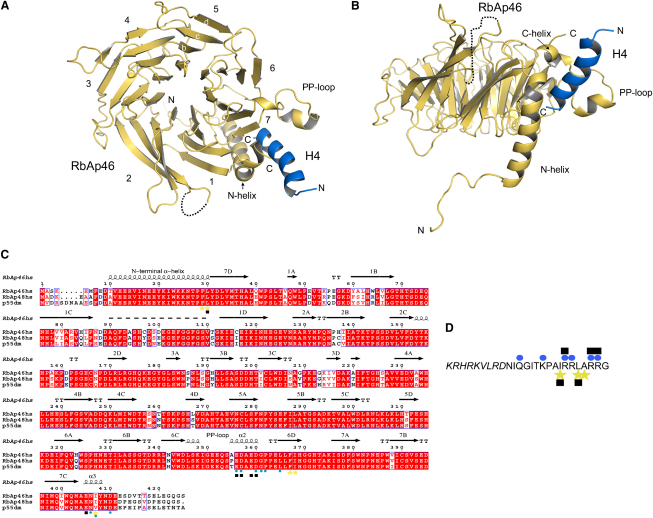
Structure of the RbAp46/Histone H4 Complex (A and B) Two different views of the structure obtained with the histone H4 peptide are shown at ∼90° to each other, as viewed from the top and side of the β-propeller structure. Residues 25–41 in histone H4 are shown in blue, whereas residues 9–410 in RbAp46 are shown in yellow. The disordered region of RbAp46 for which the electron density is unclear is shown as a dotted loop. The “Native 2” structure (PDB code: 3CFS; Tables S1 and S2) and the program PyMol (DeLano, 2002) were used to make this and the following figures. (C) Structure-based sequence alignment of the RbAp46, RbAp48, and p55 histone chaperones. The alignment is numbered as for human RbAp46. Sequences are labeled by species name (Hs, *Homo sapiens*; Dm, *Drosophila melanogaster*). The secondary structure is indicated by arrows for β strands and coils for α helices, whereas the positions of the seven blades in the β-propeller structure of RbAp46 are indicated by the numbers and letters above the β strands. Yellow stars and blue circles under the alignment indicate key residues involved in hydrophobic or hydrophilic/charged interactions with histone H4, respectively. Black squares indicate residues that were mutated. (D) Histone H4 residues (16–41) labeled as in (C). Residues in italics are not observed in the structure.

Earlier studies have shown that residues 15–41 of histone H4 are sufficient for recognition by either RbAp46 or RbAp48 (Verreault et al., 1998). We have solved the crystal structures of two human RbAp46/histone H4 complexes at high resolution (Figure 1). One complex contains a synthetic peptide encompassing residues 16–41 of histone H4, and the second includes the N-terminal tail of recombinant histone H4 (residues 1–48); these crystallized in different space groups (see Table S1 available online). Crystals were obtained of selenomethionine-substituted RbAp46 in complex with the shorter H4 peptide. The structure was solved using single-wavelength anomalous dispersion (SAD) data. The resulting model was used in molecular replacement calculations to solve the native structures. In the selenomethionine derivative, two RbAp46/H4 complexes occupy the asymmetric unit, whereas in the native structure with H4 1–48, there is only one. The crystallographic data thus provide three independently determined structures of the complex, representing different lattice environments. The three structures are essentially the same (Figure S1, Table S2) and should contain all of the important features illustrating how histone H4 binds to RbAp46.

**Figure 2:**
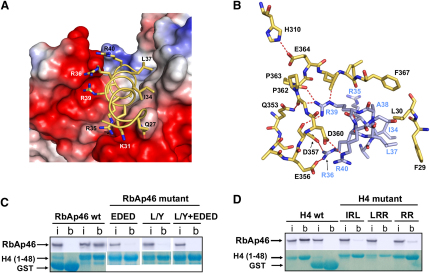
Histone H4 Recognition by the RbAp46-Binding Pocket (A) Electrostatic surface potential of RbAp46 contoured and color coded at −91 kT (red) and +91 kT (blue). The potential was calculated and displayed with the program PyMol (DeLano, 2002). The histone H4 peptide is shown as a stick model. The histone H4-binding pocket in RbAp46 is mainly formed by the negatively charged PP loop (which terminates in Pro-362 and Pro-363) and a hydrophobic surface on the N-terminal α helix (helix 1). The interaction of histone H4 residues (Gln-27, Lys-31, Ile-34, Arg-35, Arg-36, Leu-37, Arg-39, and Arg-40) is shown. (B) Detailed view showing the interactions of the hydrophobic Ile-34 and Leu-37 histone H4 residues with Phe-29 and Leu-30 in helix 1 of RbAp46, as well as the positively charged Arg-36, Arg-39, and Arg-40 histone H4 residues with the backbone carbonyl groups in the PP loop and a cluster of acidic residues (Glu-356, Asp-357, and Asp-360) in RbAp46. (C) Site-directed mutagenesis of RbAp46 in either the charged PP loop (E356Q + D357N + E359Q + D360N), the hydrophobic surface of helix 1 (L30Y), or both simultaneously all disrupt the interaction with histone H4 in pull-down experiments with GST-histone H4 1–48. (D) In reciprocal experiments, mutation of histone H4 residues interacting with either the charged PP loop (R39V + R40N), or of residues interacting with both helix 1 and the charged PP loop (I34T + L37D + R35S) and (L37D + R39V + R40N) also disrupt the binding. In both (C) and (D), the top panel shows an autoradiogram illustrating the amount of ^35^S-labeled RbAp46 pulled down in each experiment, whereas the lower panel shows a Coomassie blue-stained gel indicating the amount of either GST or GST-histone H4 (1–48) used. In each experiment, the input lane contains 30% of the ^35^S-labeled RbAp46 protein used in each of the pull-down assays. The experiments were carried out in 300 mM NaCl, 20 mM Tris (pH 8.0), 5 mM DTT, and 0.1% (v/v) NP-40.

Similar to other WD-40-repeat proteins, e.g., WDR5, which binds to the N-terminal tail of histone H3 (Couture et al., 2006; Ruthenburg et al., 2006; Schuetz et al., 2006), RbAp46 has a seven-bladed β propeller fold (Figures 1A and 1B; Figure S2). The structure is well defined, except for the N terminus (residues 2–8), a segment comprising residues 90–109, and the C terminus (residues 409–425), for which the electron density is unclear due to structural disorder. The structure of RbAp46 comprises three distinctive regions: a protruding N-terminal α helix (Asp-9 to Pro-28), which rests on the edge of blade seven; the seven blades of the β propeller (Tyr-31 to Ala-404); and a short C-terminal α helix (Glu-405 to Asn-409), which sits on top of and extends the N-terminal α helix (see Figure 1). RbAp46 also contains an unusual negatively charged loop (residues Ser-347 to Glu-364), inserted into blade six, which terminates in a Pro-362/Pro-363 sequence; we will refer to this as the PP loop. The conformation of the PP loop is stabilized by intramolecular hydrogen bonds, e.g., between the side chain of Glu-364 and the side chain of His-310, and the side chain of Gln-353 and the main chain carbonyl of Gly-361, as well as by interactions with histone H4 (Figure 2B; Figure S3). The H4 peptide adopts an α-helical conformation that is similar in all three structures, except that somewhat more residues are ordered in the RbAp46/histone H4 peptide structure. In the complex with the peptide (residues 16–41), we observe residues 25–41, whereas for the recombinant fragment of H4 (residues 1–48), residues 27–41 are well defined (Figure 1D). RbAp46 and histone H4 form extensive interactions mediated by hydrogen bonds, salt bridges, and van der Waals contacts. The protein-protein interaction buries 716 A^2^ of exposed surface area at the interface of RbAp46 and histone H4.

### Histone H4 Recognition by RbAp46

Whereas histone H3 interacts with one of the faces of the β propeller in the WDR5 protein, we find that histone H4 binds RbAp46 in a pocket on the side of the doughnut-shaped β propeller structure (Figures 1A and 1B; Figure S2). This pocket is formed between the N-terminal α helix and the PP loop. The structural elements of this pocket are unique to RbAp46 compared to other seven-bladed WD-40 β-propeller structures; in particular, this pocket is entirely absent in WDR5 (Figure S2).

The C terminus of the amphipathic histone H4 peptide (corresponding to helix 1 in the histone fold) interacts with complementary hydrophobic and charged patches in the RbAp46-binding site (Figure 2A). Residues Ile-34, Leu-37, and Ala-38 on one side of the histone H4 peptide interact with a hydrophobic patch on RbAp46 comprising residues Phe-29, Leu-30, Phe-367, Ile-368, and Ile-407 (Figure 2B; Figure S3). Through an extensive network of hydrogen bonds and salt bridges the hydrophilic face of the histone H4 helix interacts with RbAp46; H4 residues Gln-27, Lys-31, Arg-35, Arg-36, Arg-39, and Arg-40 interact with RbAp46 residues Glu-356, Asp-357, Asp-360, Gly-361, Pro-362, Leu-365, Asn-406, Ile-407, and Asp-410 (Figure 2B; Figure S3). Arg-39 and Arg-40 in the histone H4 peptide appear to play a particularly crucial role in the interaction with RbAp46. The side chain of Arg-39 forms hydrogen bonds with the main chain carbonyls of Leu-365, Gly-361, Pro-362, and Asp-357 in RbAp46. Arg-40 interacts with the side chains of Glu-356 and Asp-360 (Figure 2B; Figure S3). Histone H4 Arg-35 is another key residue, interacting with the C-terminal α helix of RbAp46 through both main chain and side chain hydrogen bonds (Figure 2; Figure S3). The key residues that are involved in the interaction with histone H4 and in defining the protein fold are identical in RbAp46, RbAp48, and *Drosophila* p55 (Figure 1C), and we conclude that all three of these proteins form the same interactions with histone H4.

Based on the structure, we designed site-directed mutants in both RbAp46 and histone H4 to disrupt the interaction between the two proteins in binding assays. Mutations in RbAp46 that alter either the hydrophobic patch, the charged PP loop, or the C-terminal helix, as well as both charged and hydrophobic side chains in histone H4 helix 1 (Figures 2C and 2D), all disrupted the interaction to various degrees. These results confirmed that RbAp46 interacts with histone H4 in solution in a similar manner to that which we observe in the crystals. We conclude that both the N- and C-terminal α helices and the PP loop in the RbAp46 pocket all make important contributions to the recognition of histone H4.

### Interaction of Histones H3 and H4 with RbAp46

**Figure 3:**
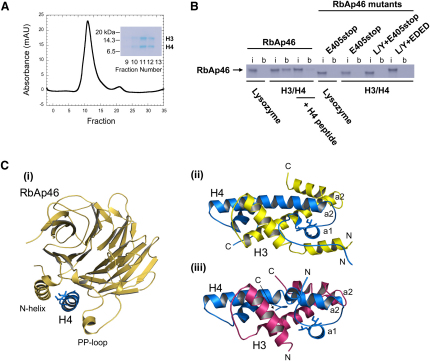
Interaction of Histones H3 and H4 with RbAp46 (A) Analytical size-exclusion chromatography of the recombinant histone H3/H4 complex used in the biochemical experiments described in this paper. The inset panel shows a Coomassie blue-stained 4%–12% NuPAGE gel used to analyze the fractions. In the conditions used here (2 M NaCl and 20 mM HEPES [pH 7.5], on a Superdex 75 PC3.2/30 column), histones H3 and H4 are present as tetramers, but at lower ionic strengths (as used in the binding experiments) these dissociate to form a mixture of dimers and tetramers (Banks and Gloss, 2003). (B) Pull-down of either wild-type or mutant RbAp46 by histones H3 and H4 crosslinked to DynaBeads, in the absence or presence of the N-terminal histone H4 peptide (residues 16–41). (The experiments were carried out as described in Figure 2. The positively charged lysozyme protein was also crosslinked to beads in separate experiments and was used as a negative control.) (C) Comparison of the interactions of Ile-34, Leu-37, and Ala-38 in helix 1 of histone H4 with (i) the N-terminal helix of RbAp46 in the RbAp46/histone H4 peptide structure, (ii) α helices 2 of histone H3 and H4 in one (of the two) H3/H4 dimer in the nucleosome core particle (Davey et al., 2002; PDB code: 1KX5), and (iii) α helices 2 of histone H3 and H4 in the ASF1-histone H3/H4 complex (English et al., 2006; Natsume et al., 2007; PDB code: 2HUE). In (i), (ii), and (iii), the view is down the axis of helix 1 of histone H4. Because similar contacts are made between histones H3 and H4 in the complex with ASF1 and in both copies of histones H3 and H4 in the nucleosome core particle, it is likely that isolated histones H3 and H4 also interact with each other in a similar manner. Histone H4 is colored blue in all three structures, whereas histone H3 is yellow in the nucleosome core particle and pink in the ASF1 complex.

Previous biochemical experiments, in which RbAp46 fused to glutathione-S-transferase (GST) was used in binding assays with histone H3/H4 dimers/tetramers, suggested that RbAp46 can bind to the histone H3/H4 complex (Verreault et al., 1998). To address whether histones H3 and H4 bind RbAp46 in a similar manner to that of the N-terminal histone H4 peptide, we first carried out competition experiments for binding to RbAp46 (Figure 3). In these experiments, histones H3 and H4 were crosslinked to beads, and then used to pull-down RbAp46 in the absence or presence of the N-terminal histone H4 peptide (residues 16–41). In the absence of the H4 peptide, but not in its presence, RbAb46 was efficiently pulled down (see Figure 3B). Mutations in the RbAp46 histone H4-binding pocket also disrupted binding to the beads. These experiments suggest that histones H3 and H4 interact with RbAp46 through the same interface as the N-terminal histone H4 peptide.

However, when the structure of histone H4 in the RbAp46 complex is compared with histone H4 in either the ASF1-histone H3/H4 complex (English et al., 2006; Natsume et al., 2007) or in the nucleosome core particle (Davey et al., 2002), it is clear that helix 1 of histone H4 adopts a significantly different conformation. The structure shows that when histones H3 and H4 bind to RbAp46, histone H4 must unfold such that helix 1 is released from its interactions with the long helix 2 of both histones H3 and H4, thereby freeing it to engage RbAp46. In particular, the residues of histone H4 helix 1 (Ile-34, Leu-37, and Ala38) that interact with RbAp46 form alternative interactions with residues in helix 2 of both histones H3 and H4 (Figure 3C). Therefore, the interactions of helix 1 in histone H4 with helix 2 in histones H3 and H4 cannot occur simultaneously with contact to either RbAp46 or RbAp48.

The finding that histone H4 must unfold partially to interact with RbAp46 raises the question of how binding of histones H3 and H4 might be accommodated? One possibility is that an unfolded H3/H4 dimer (or tetramer) can bind to RbAp46 or RbAp48. However, an alternative possibility is that histone H3 is jettisoned to yield a complex between RbAp46 and histone H4 alone. This would be consistent with previous experiments that suggest that the H3/H4 tetramer interface, and the H3/H4 histone fold, are unstable at moderate ionic strengths (Banks and Gloss, 2003).

**Figure 4:**
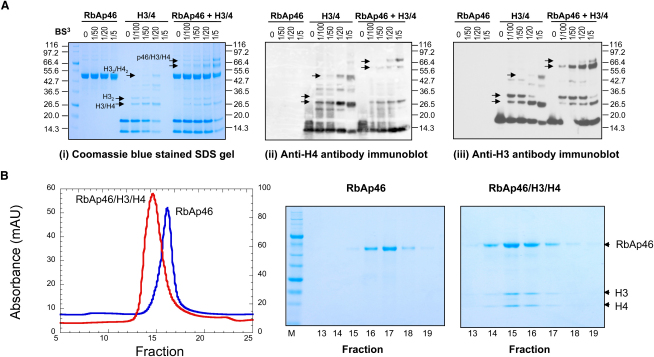
Interaction of Histones H3 and H4 with RbAp46 (A) Comparison of chemical crosslinking of histones H3 and H4 in the absence and presence of RbAp46. Three different 4%–12% NuPAGE gels were either (i) stained with Coomasie blue or blotted with (ii) anti-histone H4 or (iii) anti-histone H3 antibodies. By themselves, histones H3 and H4 appear to form a H3/H4 heterodimer (∼25 kDa), an H3_2_ homodimer (∼30 kDa), and an H3_2_/H4_2_ heterotetramer (∼50 kDa), indicated by arrowheads. In the presence of RbAp46, two bands, which both contain histones H3 and H4 (also indicated by arrowheads), can be seen in positions roughly corresponding to the interaction of RbAp46 with histone H3/H4 dimers and tetramers (∼65–75 kDa). Control crosslinking experiments of RbAp46 with positively charged lysozyme were also carried out in the same conditions, but yielded no crosslinked species (data not shown). (In the H4 Western blot, staining with the anti-histone H4 antibody appears to be inhibited by the presence of histone H3, leading to white bands. In addition, at the higher concentrations of crosslinker, extensive crosslinking of the proteins results in the formation of large complexes that do not enter the gel—e.g., the band corresponding to the H3_2_ complex becomes less intense. The antibodies may also recognize their epitopes less efficiently—as has been noted previously [Banks and Gloss, 2003].) (B) Analytical size-exclusion chromatography, on an Ettan LC system (GE Healthcare), of either 25 μM RbAp46 (blue trace) or 25 μM RbAp46/H3/H4 (red trace), in 200 mM KCl and 20 mM HEPES (pH 7.4), at 4°C on a Superdex 200 PC3.2/30 column. The fractions were collected and analyzed on 4%–12% NuPAGE gels.

The aforementioned pull-down experiments, in which RbAp46 was pulled-down by histones H3 and H4 linked to beads, suggest that RbAp46 may be able to interact with the histone H3/H4 complex. However, histone H3 could be jettisoned from the complex during the interaction. To explore this point, we carried out chemical crosslinking experiments to address the possibility that the interaction of RbAp46 with histone H4 might disrupt the interaction with histone H3 in histone H3/H4 dimers (or tetramers). The crosslinked species were identified by immunoblot. Consistent with previous work, with either glutaraldehyde (Banks and Gloss, 2003) or dimethyl suberimidate (Thomas, 1989) chemical crosslinking, we found that the major crosslinked H3/H4 species migrates as the heterodimer. Smaller amounts of homodimeric (H3_2_), trimeric (H3_2_/H4), and tetrameric (H3_2_/H4_2_) complexes (as noted previously) are also observed (see Figure 4A).

In the presence of RbAp46, crosslinked species of RbAp46 with both histones H3 and H4 could be detected. The presence of both histones H3 and H4 was confirmed in immunoblots probed with antibodies to histones H3 and H4 (Figure 4A, panels [ii] and [iii]). In these experiments two bands that migrate more slowly than the RbAp46 protein by itself were detected. The crosslinked protein complex migrating immediately above RbAp46 was recognized strongly by the anti-histone H3 antibody and more weakly by the anti-histone H4 antibody. The crosslinked protein complex migrating at higher molecular weight, however, although present at lower amounts, was recognized equally well by both antibodies. The resolution in this region of the gel does not allow us to identify the species present in the two complexes, but given that the major crosslinked H3/H4 species (by itself) is the heterodimer, with smaller amounts of the trimeric and tetrameric complexes (see above), we would expect that similar proportions of these different complexes would also crosslink to RbAp46. In other words, we would expect that the lower band corresponds to the dimeric H3/H4 complex crosslinked to RbAp46, and that the higher band corresponds to either the trimeric (H3_2_/H4) or the tetrameric (H3_2_/H4_2_) complexes crosslinked to RbAp46. It is not clear why histone H4 is recognized much less efficiently by the antibody in the lower band (the presumed RbAp46/H3/H4 complex; compare panels [ii] and [iii] in Figure 4A), but because histone H4 epitopes may be recognized differently in the different complexes, it is perhaps not unexpected. In particular, if the higher M_r_ band corresponds to RbAp46 bound to the tetramer (H3_2_/H4_2_), then a second histone H4 molecule will be available for recognition by the antibody, perhaps explaining the more equal staining of that band by the two antibodies. Regardless, the important point is that both histones H3 and H4 are found in the same complex(es) with RbAp46, demonstrating that histone H3 is not jettisoned from the H3/H4 complex when histone H4 binds RbAp46.

To support the results of the crosslinking experiments, and to further demonstrate that histone H3 is not jettisoned from the complex, we analyzed a (1:1) molar ratio of RbAp46 and the histone H3/H4 dimer by using analytical size-exclusion chromatography. These experiments showed that RbAp46 now elutes with histones H3 and H4 as a higher molecular complex in comparison to the position it elutes by itself. Importantly, histones H3 and H4 are present in equal amounts, directly demonstrating that histone H3 is present in the complex (Figure 4B). Although there is some overlap between the positions at which RbAp46 and the RbAp46/H3/H4 complex elute, the results also suggest that all of the RbAp46 forms a complex with H3/H4 when the two are mixed in a 1:1 ratio.

Next, we used the intrinsic tryptophan fluorescence of RbAp46 (Trp23 interacts with histone H4 in the binding pocket) to measure its affinity of binding to histone H3/H4 dimers (or tetramers). RbAp46 binds to histones H3 and H4 with an apparent K_D_ of 0.9 μM. By comparison, the K_D_ for the binding of the N-terminal histone H4 peptide is 1.1 μM. The similarly high affinity for the interaction of RbAp46 with either histones H3 and H4 or the histone H4 peptide was corroborated by surface plasmon resonance (SPR) experiments (both K_D_s were ∼1 μM). In summary, the gel-filtration experiments directly show that the histone H3/H4 complex can interact with RbAp46. The similar K_D_s for RbAp46 binding to histones H3 and H4 and the N-terminal histone H4 peptide, as measured by fluorescence spectroscopy and SPR experiments, show that there is little barrier to the unfolding of H4 helix 1 in the H3/H4 complex. The results suggest that the structure of the histone H3/H4 complex has a hitherto unexpected flexibility that allows it to readily interact with RbAp46.

## Discussion

Our crystallographic and biochemical data indicate that H4 binds to a groove on the edge of the β propeller fold, and that this engages the C-terminal portion of the first helix of the histone fold. The importance of key contacts is corroborated by site-directed mutagenesis. We anticipate that this mode of binding is conserved in the p55 and RbAp48 homologs since the determinants of specificity are conserved in their amino acid sequences. Computational optimal desolvation analysis (Cheng et al., 2007) and docking experiments suggest that partner proteins, e.g., HAT1, will likely bind on one face of the RbAp46 or RbAp48 β-propeller (Figure S4). The mode of histone H4 binding that we observe presents the flexible amino terminus of H4 in the direction needed such that it can engage the active site of such partner proteins.

As mentioned in the introduction, RbAp46 and RbAp48 enhance the activities of many protein complexes involved in chromatin remodeling and the covalent modification of nucleosomal histones. The release of helix 1 from the histone fold of histone H4 might be important for making the N-terminal tail more accessible as a substrate for the catalytic subunits of histone-modifying complexes. Conceivably, the partial unfolding of histone H4 when bound to RbAp46 and RbAp48 chaperones might also facilitate the action of ATP-dependent chromatin-remodeling complexes.

Our biochemical experiments show that histone H3/H4 dimers/tetramers can bind to RbAp46. In the crystal structures of ASF1 and the nucleosome core particle, histones H3 and H4 retain the same tertiary and quaternary contacts. These same contacts cannot be supported in a structurally rigid docking of a histone H3/H4 dimer to the surface of RbAp46 if the H4 were to interact in the mode observed in our crystal structures. Taken together, these results suggest that histones H3 and H4 must undergo a conformational change in order to bind RbAp46. We anticipate that this structural transition in histones H3 and H4 may be somewhat dynamic, even in the absence of RbAp46, and may be linked to the processes of histone recognition, chemical modification and nucleosome assembly catalyzed by the machinery of which RbAp46 and RbAp48 are key components.

For a long time it has been widely assumed that histones H3 and H4 exist as H3_2_/H4_2_ tetramers in vivo when they are not bound to chromatin. However, two lines of evidence have drastically changed this view. First, epitope-tagged histone H3 does not copurify with endogenous histone H3 in chromatin-assembly complexes (Tagami et al., 2004). In addition, the crystal structures of ASF1 bound to H3/H4 demonstrated that the ASF1 histone chaperone can only bind to a histone H3/H4 dimer because ASF1 occludes the surface of H3 that is necessary for tetramer formation (English et al., 2006; Natsume et al., 2007). Stepwise formation of H3_2_/H4_2_ tetramers by consecutive deposition of two H3/H4 dimers has thus been proposed for both replication-coupled and replication-independent nucleosome assembly (Agez et al., 2007; Antczak et al., 2006; English et al., 2006; Natsume et al., 2007). The RbAp48 histone chaperone, which is highly homologous to RbAp46, is a member of the CAF-1 complex that cooperates with ASF1 in replication-dependent nucleosome assembly (Tyler et al., 1999; Verreault et al., 1996). ASF1 interacts mainly with the C-terminal helix of histone H3 that is located on the opposite face of the H3/H4 dimer from the unfolded helix 1 in histone H4 to which RbAp46 and RbAp48 bind. The results we report here suggest how the RbAp48 subunit in CAF-1 might accept an H3/H4 dimer from ASF1 for nucleosome assembly. In particular, one can imagine that the flexibility in histone H4 might allow RbAp48 and ASF1 to simultaneously interact with the same H3/H4 dimer, allowing the two histone chaperones to coordinate the supply of H3/H4 dimers during chromatin assembly.

Because RbAp46 binds to the opposite side of the H3/H4 complex from that which forms the H3/H3 homodimer interface in H3_2_/H4_2_ tetramers, the structure as well as the crosslinking experiments also suggest that RbAp46 and RbAp48 should be able to bind both H3/H4 dimers and H3_2_/H4_2_ tetramers equally well. Moreover, the relative amounts of the two bound species may simply depend on the propensity of H3 to homodimerize under the conditions involved. In the absence of ASF1, the ability of RbAp46 and RbAp48 to interact with both H3/H4 dimers and H3_2_/H4_2_ tetramers may be important for their functions in HDAC and methylase complexes, as well as in nucleosome-remodeling factors.

## Experimental Procedures

### Protein Expression, Purification, and the Generation of Mutants

Recombinant histone H4 and RbAp46 proteins were expressed and purified as described previously (Verreault et al., 1998; Berger et al., 2004). Detailed procedures for the generation of the RbAp46/histone H4 peptide and recombinant histone H4 complexes can be found in the Supplemental Data. Recombinant full-length (*Xenopus lavis*) histones H3 and H4 were expressed, purified, and refolded as described (Luger et al., 1997). For the binding experiments, histones H3/H4 and lysozyme (Sigma) were crosslinked to DynaBeads (Dynal Biotech) according to the manufacturer's recommendations. RbAp46 and histone H4 mutants were generated by using the QuikChange Site-Directed Mutagenesis kit (Stratagene). The RbAp46 mutants were expressed by using a Rabbit Reticulocyte Cell Free system (Promega).

### Biochemical Crosslinking

RbAp46/H3/H4 crosslinking was performed by using the BS^3^ crosslinker (Pierce) in 1.1 M NaCl, 20 mM HEPES (pH 7.5), and 10 mM DTT. Freshly prepared BS^3^ stock solution, 3% (w/v), was diluted 1/5, 1/10, 1/20, 1/50, and 1/100 with distilled water, and 1 μl of the resulting solution was mixed with 10 μl of ∼10 μM RbAp46, recombinant histone H3/H4, or RbAp46/H3/H4 prior to incubation at room temperature for 30 min. Crosslinking was stopped by adding 1 μl of 1 M Tris (pH 8.0) to each reaction and incubating for 15 min at room temperature. The proteins were analyzed on separate 4%–12% NuPAGE gels, one for Coomassie blue staining and one for each Western blot with either anti-histone H3 (Abcam) or anti-histone H4 (A.V.) antibodies.

### Fluorescence Spectroscopy

Binding of increasing concentrations of either recombinant histones H3 and H4 or the N-terminal histone H4 peptide (residues 16–41) to 45 nM RbAp46 in 50 mM Tris-HCl (pH 7.5), 200 mM NaCl, and 5 mM DTT was measured by fluorescence emission at 435 nm with a Perkin-Elmer LS55 spectrofluorimeter by using an excitation wavelength of 295 nm with an excitation and emission band pass of 10 nm at 25 (±0.1)°C. The change in fluorescence was corrected by titrations with buffer alone and measurement of the fluorescence of H3/H4 in the absence of RbAp46. Using a single binding-site model, in which the concentration of the RbAp46/ligand complex is directly proportional to the change in fluorescence (Δ*F*), the corrected data were fitted to the following equations:

(1) $$\Delta F=\frac{\Delta F_{\max}\left[L\right]}{K_{d}+\left[L\right]},$$

(2) $$\Delta F=\Delta F_{\max}-K_{d}\cdot \frac{\Delta F}{\left[L\right]},$$

where K_D_ is the dissociation constant and [L] is the concentration of ligand.

### Surface Plasmon Resonance

Amine crosslinking was used to attach recombinant histones H3 and H4 to the surface of a CM5 sensor chip to a level of 1700 RU (GE Healthcare) by following the supplied protocol. Measurements were made by using chips equilibrated in 10 mM HEPES (pH 7.4), 150 mM NaCl, and 0.0005% (v/v) Surfactant P20 (GE Healthcare). The volume of RbAp46 analyte injected was 90 μl at a rate of 30 μl/min. The concentrations of analytes were varied to test for mass-transport effects. The signal from the protein-coupled cell was compared to that from the blank reference cell. For measurement of binding of the N-terminal histone H4 peptide, RbAp46 was instead crosslinked to the CM5 sensor chip.

### Crystallization and Structure Refinement

Native crystals of the RbAp46/recombinant histone H4 (1–48) complex were grown at 4°C by vapor diffusion in hanging drops by using 18% PEG 8000, 0.2 M calcium acetate, and 0.1 M sodium cacodylate (pH 6.4) as the precipitant. Native crystals of the RbAp46/synthetic histone H4 peptide (residues 16–41) complex were grown at 4°C by vapor diffusion in hanging drops by using 9% w/v PEG 6000, 0.2 M calcium acetate, and 0.1 M sodium cacodylate (pH 6.3) as precipitant. Similarly, crystals of the selenomethionine derivative of RbAp46 in complex with the H4 peptide (residues 16–41) were grown at 4°C by using 13% w/v PEG 2000, 0.2 M calcium acetate, and 0.1 M sodium cacodylate (pH 6.3) as precipitant. Crystals were harvested and cryoprotected by serial transfer through a series of solutions containing increasing amounts of glycerol (to 22.5%). Crystals were then flash cooled in liquid nitrogen to 100 K. Crystals of the native RbAp46/H4(1–48) complex belong to the orthorhombic P2_1_2_1_2_1_ space group with unit cell dimensions of a = 44.67, b = 85.73, and c = 117.72 Å. The native and selenomethionine RbAp46/H4(16–41) crystals belong to the monoclinic P2_1_ space group with pseudo tetragonal unit cell dimensions of a = 108.66, b = 44.79, c = 109.59, β = 90.71° and a = 109.04, b = 44.72, c = 109.35Å, β = 91.13°, respectively (see Table S1).

Native X-ray diffraction data were collected at stations ID23-2 and ID29 (ESRF), and single-wavelength anomalous dispersion (SAD) data from the selenomethionine crystal were collected at station ID14-4 (ESRF) (Table S1). The Native data were processed in HLK2000 and scaled in SCALEPACK2000 (Otwinowski and Minor, 1997). The SAD data were processed by using MOSFLM, SCALA, and TRUNCATE (CCP4, 1994). Heavy-atom sites were identified and iteratively improved by using HKL2MAP (Sheldrick, 1990), AUTO-SHARP (Vonrhein et al., 2007), and PHENIX (Terwilliger et al., 2008), yielding 15 selenium and 2 arsenate sites. After solvent flattening, density modification, and NCS averaging, the auto-build mode of PHENIX produced a model with 657 main chain and 315 side chain fragments out of a total of 888 residues. The map produced was of sufficiently high quality to permit confident interpretation, and tracing of the backbone and side chains, which was completed using COOT (CCP4, 1994). Restrained, NCS, and TLS refinement of the model was carried out by using REFMAC5 (CCP4, 1994). The final refined structure of the selenomethionine derivative (Se-Met RbAp46/H4(16–41) (Peak); see Table S1) contains RbAp46 residues 1–89 and 110–410, together with histone H4 residues 25–41. This structure was used as a search model in molecular replacement calculations to determine both native structures (Native 1 and Native 2; Table S1). In the 2.4 Å structure of native RbAp46/H4(1–48), the final model contains RbAp46 residues 1–89 and 111–410, together with histone H4 residues 27–41 (RbAp46/H4 (Native 2); Table S1). The stereochemistry of the models was validated by using SFCHECK and PROCHECK (CCP4, 1994).

## Accession Numbers

The coordinates have been deposited in the PDB with codes 3CFV and 3CFS for Se-Met RbAp46/H4(16–41) (Native 1) and RbAp46/H4 (Native 2), respectively.

## Supplemental Data

Document S1. Supplemental Experimental Procedures, Two Tables, and Four Figures
