# Supplementary material for: Structural Basis for the Recognition of Histone H4 by the Histone-Chaperone RbAp46
Source: Structure. 2008 Jul 9;16(7):1077–85. doi: 10.1016/j.str.2008.05.006 (PMC2572730; doi:10.1016/j.str.2008.05.006)

Structure *16*

Supplemental Data

Structural Basis for the Recognition of Histone H4 by the Histone-Chaperone RbAp46

Natalia V. Murzina, Xue-Yuan Pei, Wei Zhang, Mike Sparkes, Jose Vicente-Garcia, J. Venkatesh Pratap, Stephen H. McLaughlin, Tom Rolef Ben-Shahar, Alain Verreault, Ben F. Luisi, and Ernest D. Laue

Supplemental Experimental Procedures

Expression and Purification of RbAp46 in a Complex with Recombinant Histone H4

The recombinant N-terminal tail (residues 1-48) of histone H4 was expressed and purified as described previously (Verreault et al., 1998). RbAp46 was also expressed in insect cells using the pPK13 baculovirus as described (Verreault et al., 1998). For RbAp46 affinity purification the pre-cleared cell extract was applied to a column containing GST-H4 (1-48) bound to glutathione agarose. After extensive washing with 20 mM Tris pH 7.5 containing 100 mM NaCl, 2 mM DTT and 0.2% (v/v) NP-40, the RbAp46/H4 (1-48) complex was released by cleavage of histone H4 from GST with thrombin. Mass spectrometry showed that the histone H4 in the complex was degraded, with residue 16 being the N-terminus of the shortest peptide. The complex was further purified on a MonoQ column, buffer exchanged into 100 mM NaCl and 20 mM Tris pH 7.5 and concentrated using a Vivaspin 6 concentrator to 13 mg/ml for crystallisation.

Expression and Purification of Native and Selenomethionine-Labelled RbAp46

His-tagged RbAp46 was expressed in insect cells by transferring a BglII-HindIII fragment of pET15b, containing the RbAp46 gene incorporated in the NdeI and BamHI sites, into the BamHI and HindIII sites of pFBDM (Berger et al., 2004). The recombinant pFBDM-p46HIS baculovirus, generated by recombination with a bacmid in DH10Bac *E.coli* (Invitrogen), was used for expression of both native and selenomethionine RbAp46 in insect cells using Sf-900 II serum-free medium (Invitrogen). RbAp46 selenomethionine derivatives were expressed in the methionine free version of the Sf-900 II media (Invitrogen), supplemented with 150 mg/ml selenomethionine. The cells were harvested at ~55 hours post-infection and the protein was purified by affinity chromatography on a Ni-NTA column (Qiagen) following standard procedures. The His-tag was removed by thrombin cleavage and the RbAp46 was

purified further on a MonoQ column (GE Healthcare), buffer exchanged into 100 mM NaCl and 20 mM Tris pH 7.5, combined with a histone H4 peptide (residues 16-41) in a 1:2 molar ratio and concentrated on a Vivaspin 3 concentrator to 13 mg/ml for crystallisation.

Table S1. Summary of the Crystallographic Data

Data sets	RbAp46/H4(16-41) (Native 1)	RbAp46/H4 (Native 2)	Se-Met RbAp46/H4(16-41) (Peak)
Space group	$p2_1$	$p2_12_12_1$	$p2_1$
Unit cell (Å) (°, degree)	a=108.66, b=44.79, c=109.59, β=90.71°	a=44.67, b=85.73, c=117.72,	a=109.03, b=44.71, c=109.36, β=91.13°
Wavelength (Å) (station)	0.873 (ID23-2)	0.9793 (ID29)	0.97932 (ID14-4)
Resolution (Å)	50-2.6	20-2.4	50-2.8
Unique reflections	33119	18615	25478
Completeness (%) (outer shell)	99.9 (99.9)	99.8 (98.0)	96.5 (96.5)
Anomalous Completeness (%) (outer shell)	---	---	96.9 (94.6)
R_{merge} (outer shell)	0.143 (0.731)	0.130 (0.468)	0.129 (0.465)
R_{anom} (outer shell)	---	---	0.055 (0.156)
Data redundancy	3.7	7.0	8.1
Anomalous data redundancy	---	---	4.1 (4.1)
Average I/σ (outer shell)	18.5 (3.3)	16.0 (1.9)	16.8 (5.0)
**Refinement**			
Resolution (Å)	50-2.6	20-2.4	50-2.8
Number of Atoms	6915	3413	6800
Number of reflections	31416	17375	25478
Completeness (%)	99.6	99.95	99.51
$R(_{free+work})$ (%)	18.01	16.91	---
R_{work} (%)	17.6	16.51	18.31
R_{free} (%) (5% of reflections)	25.3	24.27	26.69
Mean B-factor ($Å^2$)	48.94	32.83	46.16
R.M.S.D bond length (Å)	0.007	0.009	0.011
R.M.S.D bond angle (degree)	1.02	1.202	1.12
Ramachandran (%) (core, allowed, general, disallowed)	86.6, 12.7, 0.7, 0.0	88.6, 10.2, 1.2, 0.0	84.3, 12.8, 1.5, 1.4

Table S2. Rmsd (Å) between the Different RbAp46/Histone H4 Structures

	Se-Met RbAp46/H4 (2.8Å)	Native 1 RbAp46/H4 (2.6Å)
RMSD for RbAp46 (7-410) from **Native 2 RbAp46/H4 (2.4Å)**	0.655	0.529
RMSD for H4 (30-41) from **Native 2 RbAp46/H4 (2.4Å)**	0.321	0.264

See following pages for figures.

Figure S1. Comparison of the Three Different Structures of the RbAp46/H4 Complex Determined in This Work

The Native 1 RbAp46/H4 (2.6 A) structure is shown in blue; the Native 2 RbAp46/H4 (2.4 A) structure is shown in yellow; and the Se-Met RbAp46Se/H4 (2.8 A) structure is shown in red.

Figure S2. Comparison of the Structure of the WDR5/Histone H3 Complex (Ruthenburg et al., 2006; Schuetz et al., 2006; PDB code 2H9L) with the Structure of the RbAp46/Histone H4 Peptide Complex Determined in This Work

The superposition highlights the presence of the unique histone H4-binding pocket in RbAp46, which is formed by the N- and C-terminal α-helices and the PP-loop. This pocket is not conserved in other 7-bladed WD-40 β-propeller proteins such as WDR5.

Figure S3. Stereo-Version of Figure 2B Showing the Interactions of the Hydrophobic Ile-34 and Leu-37 Histone H4 Residues with Phe-29 and Leu-30 in Helix-1 of RbAp46, as Well as the Positively Charged Arg-36, Arg-39 and Arg-40 Histone H4 Residues with the Backbone Carbonyl Groups in the PP-Loop and a Cluster of Acidic Residues (Glu-356, Asp-357 and Asp-360) in RbAp46

Figure S4. Prediction of Possible Protein Binding Sites on the Surface of the RbAp46/Histone H4 Complex Using Computational Optimal Desolvation Analysis (Cheng, T.M. et al., 2007)

Two different views, from the top and bottom sides of the β-propeller structure, are shown with the RbAp46 surface contoured and colour coded dark blue, light blue, and green – in the order of the strength of the prediction. The histone H4 peptide is shown as a cartoon model.

Figure S1 – Murzina et al.,

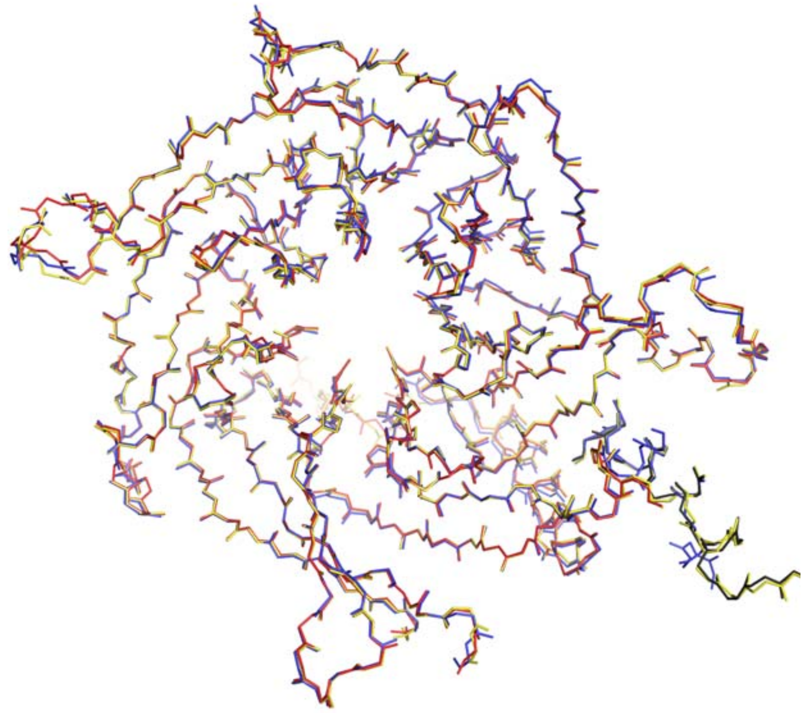

Figure S2 – Murzina et al.,

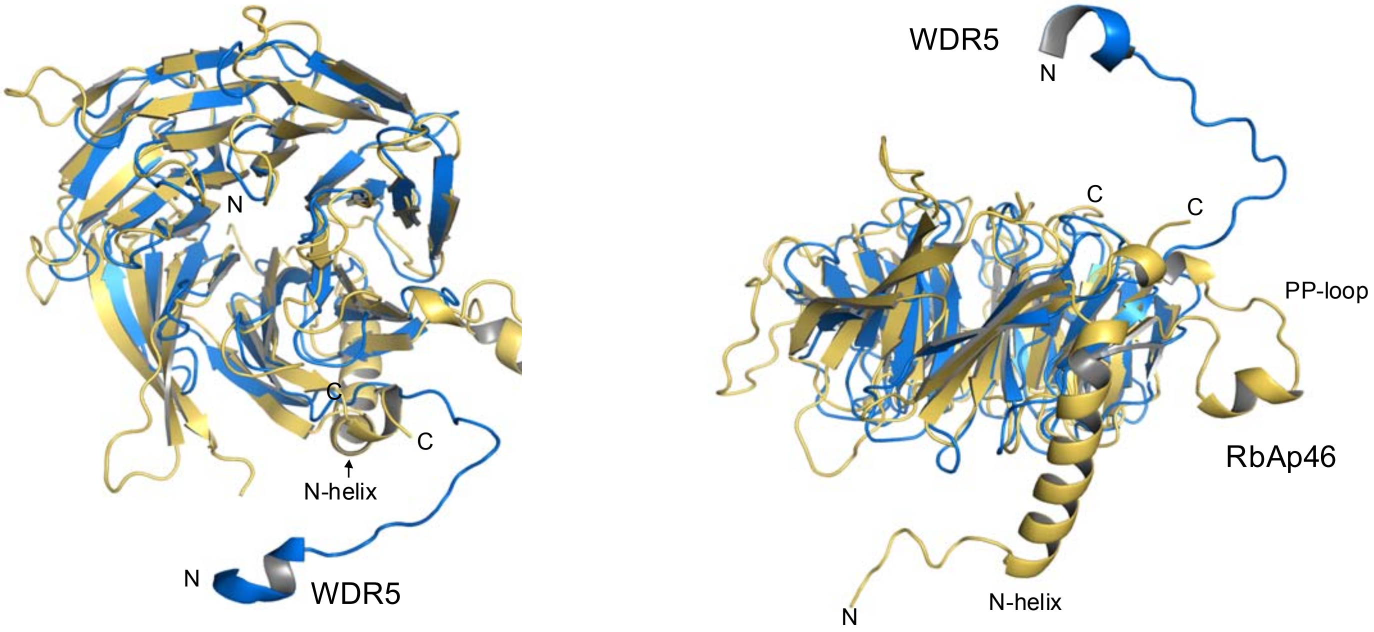

Figure S3 – Murzina et al.,

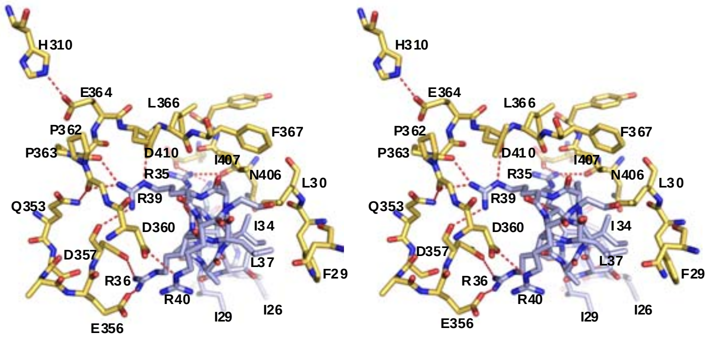

Figure S4 – Murzina et al.,

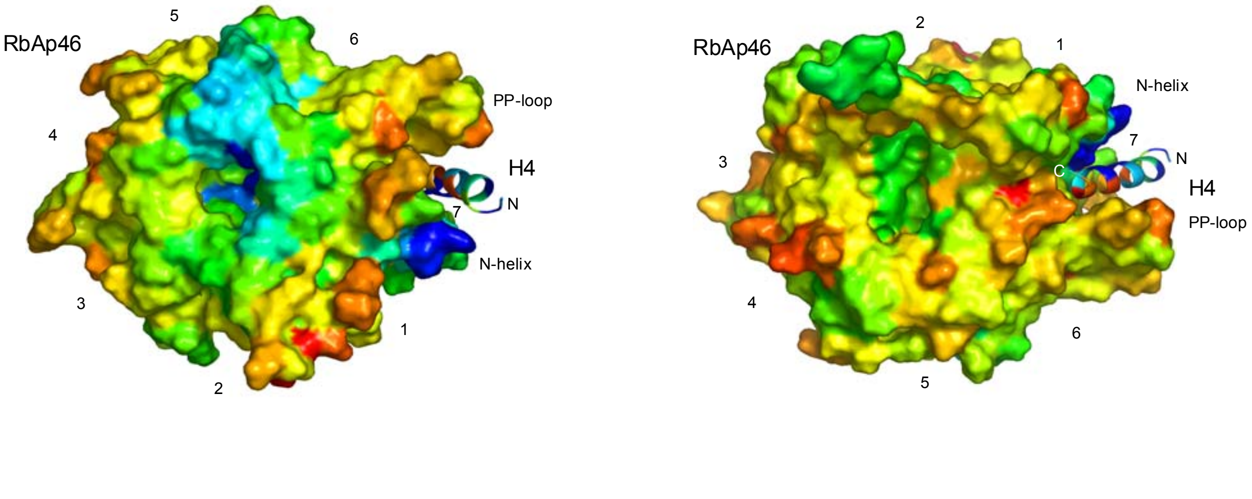

Supplement: Document S1. Supplemental Experimental Procedures, Two Tables, and Four Figures [file mmc1.pdf]
